# Influencing factors of psychological stress under the mixed teaching mode based on SPOC+PBL

**DOI:** 10.3389/fpsyg.2022.979206

**Published:** 2022-09-06

**Authors:** Guorong Shen, Yide An

**Affiliations:** ^1^School of Foreign Languages, Henan University of Technology, Zhengzhou, Henan, China; ^2^Department of Physical Education, Tianjin Medical University, Tianjin, China

**Keywords:** blended teaching, psychological stress model, SPOC + PBL, analysis of influencing factors, design of new teaching mode

## Abstract

In the teaching process, teachers and students are under different psychological pressures. Especially under the influence of the epidemic, many teaching modes have been transformed from traditional classroom teaching to online teaching. Under the new teaching mode, especially the promotion and application of the blended teaching mode, classroom teaching is facing new challenges, and both students and teachers are facing new psychological pressures. If the psychological pressure is not resolved, the classroom effect will be even worse. Therefore, the purpose of this paper is to analyze the source of the influencing factors of psychological stress under the new teaching mode, which is a rarely involved direction in the teaching field, and is of great significance for the promotion and application of the new teaching mode. Aiming at the blended teaching model, this paper focuses on the SPOC+PBL blended teaching model, designs a new teaching model of “English-Chinese translation and interpretation”, and studies the sources of pressure under the new model. Aiming at the influencing factors of psychological pressure, a psychological pressure model is established to explore the influence of six factors on psychological pressure. The experimental results of this paper show that the factor loadings of all variables are greater than 0.5, indicating that “self-esteem strength”, “self-efficacy”, “help-seeking experience”, “psychological counseling cognitive bias”, “counseling object positioning bias” and “psychological counseling” “Resources” and “availability” have a greater impact on psychological stress.

## Introduction

The official proposal of MOOC in 2012 has not only set off a global upsurge in online education, but also brought a huge impact on traditional education. MOOC’s shared high-quality resources, diverse learning evaluations, timely learning feedback, and diverse learning support services make it uniquely different from previous online courses. With the help of third-party online learning platforms, MOOCs quickly have swept the education field into a period of rapid development. In terms of education model, it is the rise of SPOC. With the application of the new teaching mode, teachers and students need to take time to adapt to the teaching rhythm under the new mode. Due to the conflict between the new mode and the traditional teaching mode, teachers and students are inevitably under psychological pressure. Problem-driven teaching method is a learning method that takes students as the main body, takes various problems in the professional field as the starting point of learning, plans the learning content with the problem as the core, and allows students to seek solutions around the problem. At the same time, the change of teaching mode will also have a greater impact on students’ learning and teachers’ teaching, so it is necessary to explore the source of psychological pressure for the change of teaching mode.

Psychological problems have always been the focus of health scientists, especially the mental health of school teachers and students, which has received more attention from scholars. Mennis J examined the transient link between urban green space and psychological stress. Saeidi M aimed to explore the role of perceived cardiac risk factors in the prediction of psychological symptoms in cardiac rehabilitation patients (Saeidi et al., 2018). Of these, psychological stress was captured using the Geographic Ecological Momentary Assessment (GEMA), primarily in the activity space of a sample of African-American adolescents living in Richmond, Virginia (Mennis et al., 2018). Major depressive disorder (MDD) is a heterogeneous and multifactorial disease, and Liu L studied its underlying molecular mechanisms (Liu et al., 2018). Liu Y proposed a method to detect psychological stress levels, which aimed to explore the feasibility of using a single physiological signal to create a more practical alternative to current multi-physiological signal methods to detect people’s stress (Liu and Du, 2018). The purpose of Agarwal A’s study was to observe psychological stress in an auditory sample of daily wage workers in different states of India (Agarwal, 2021). It can be found that although the psychological research is relatively in-depth, there is not much data on the source of psychological stress factors brought about by the change of teaching mode.

With the rapid development of the information age, not only the learning forms are more diverse, but the learning methods are more convenient. There has also been a lot of research on new teaching models. Busto S has presented a case study of a simple yet effective technical and logical concept for the realization of a blended teaching of mathematics and its application in theoretical mechanics. His experimental results prove that in a blended teaching model, simple logical concepts are more easily accepted by students (Busto et al., 2021). Li Y has studied the mixed teaching mode of “micro-lecture” and “rain class” based on the Internet, which makes up for the insufficiency of the traditional teaching mode and improves the learning effect (Li and Qu, 2021). Taking the course “College English” as an example, Wu X discussed the relationship between blended teaching and the teaching of ideological and political courses, analyzed the teaching methods of blended teaching, and put forward suggestions for reforming the evaluation system for the two parts (Wu, 2022). Although there are many applications of blended teaching and some teaching effects can be achieved, the research of these scholars has also exposed the problems of improper depth of blended teaching, such as fragmented learning and surface reading.

Among the students surveyed, 76.47% are satisfied with the overall learning experience and the learning effect obtained; and 70.59% of the students are willing to recommend or continue to participate in the teaching courses in the form of PBL+SPOC. Therefore, it can be seen that students have high learning satisfaction and model recognition for the classroom based on SPOC+PBL. The innovation of this paper is mainly to design a model of students’ psychological stress sources for the change of teaching mode. Through the selected 6 factors, a comparative analysis of the sources of students’ stress can be made, which can clearly reflect the source of psychological stress. This is of great significance for the acquisition of the influencing factors of psychological stress in the mixed teaching mode of SPOC+PBL.

## Psychological changes under blended teaching

### Mixed teaching method of SPOC+PBL

**FIGURE 1:**
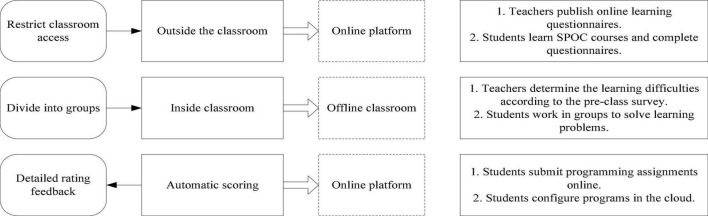
Blended teaching method.

The current SPOC teaching case is mainly set up for two types of learners, college students and students in school. It is a blended learning model that combines classroom teaching and online teaching resources. In the PBL teaching method, the problem is the core point and the source of power for the operation of teaching activities. Students are the main body of learning and a necessary condition for teaching activities. The teacher is the guide and the main condition for the development of teaching activities. SPOC+PBL is essentially a hybrid teaching model combining teaching methods and teaching resources. Due to the impact of the epidemic, Schools canceled offline teaching on a large scale and adopted online teaching, which has greatly promoted the development of online courses. In the new teaching environment, more emphasis was placed on the subject status of students (Jin, 2021; Juan, 2021). SPOC is derived from MOOC, and its essence is to recreate and reshape on the basis of the continuation of MOOC. It applies the concept of opening and sharing knowledge of MOOC resources to group users in colleges and universities, and makes use of high-quality MOOC resources and online evaluation and communication. Interaction and other functions transform traditional classroom teaching and effectively promote the deep integration of MOOC and traditional classroom teaching. It is a typical curriculum paradigm in the “post-MOOC” era. The PBL teaching method has its advantages in teaching. The advantages are as follows: first, students are highly motivated to participate in the whole process of learning; second, students learn around problems and learn new knowledge through the process of analyzing and solving problems, which helps to improve students’ learning. Comprehensive quality; third, the new knowledge and different ways of thinking that students learn from learning activities will benefit lifelong. As one of the teaching methods in colleges and universities, SPOC lacks interactivity and students’ independent thinking. Even some SPOC classes simply play video content. The shortcomings of PBL are also obvious, with students’ self-directed thinking lacking direction and too simple problems, which has made group learning lack of inquiry (Wang and Wang, 2019; Li, 2021). The SPOC+PBL blended teaching is shown in Figure 1. However, SPOC+PBL can perfectly combine the advantages of the two, giving full play to the enthusiasm of the classroom and at the same time taking more abundant resources to assist.

### Psychological pressure under the blended teaching method

The new teaching mode is bound to bring certain changes to students and teachers. From the perspective of teachers, under the new teaching mode, it is necessary to prepare new courseware, design new teaching methods and adapt to new teaching pressures; for students, SPOC +PBL courses are relatively unfamiliar, and there may be a series of psychological pressures such as anxiety. Such pressure will lead to the decline of teaching effect, so it is necessary to study the psychological model under the new teaching mode (Tan, 2021).

Under SPOC teaching, learning adopts a combination of online and offline mode, and there is less contact between teachers and students, which leads to insufficient understanding of teachers on students, so that teachers cannot teach students according to their aptitude, and cannot exert their professional abilities, resulting in poor teaching mood., resulting in increased pressure. Therefore, for SPOC teaching, it is necessary to fully understand the principles of teaching, in order to fundamentally solve the teaching pressure of teachers (Liu, 2021).

Under the PBL teaching, the classroom places more emphasis on the students’ autonomous learning ability, so that the status of teachers is lost, and teachers’ confidence in teaching and teachers’ teaching psychology will be seriously shaken. Therefore, in order to obtain a better teaching effect, it is necessary to start from the teaching process. Have a deep understanding of the principles of the new teaching model, and solve the anxiety of uncertainty from the process.

The new teaching mode is bound to bring certain changes to students and teachers (Daniels, 2011; Stefanie, 2022). From the perspective of teachers, under the new teaching mode, it is necessary to prepare new courseware, design new teaching methods and adapt to new teaching pressures; for students, SPOC +PBL courses are relatively unfamiliar, and there may be a series of psychological pressures such as anxiety. Such pressure will lead to the decline of teaching effect, so it is necessary to study the psychological model under the new teaching mode (Rui and Lianrong, 2021).

### Optimization algorithm of psychological model

In the psychological stress modeling of the new teaching mode, many different indicators and variables need to be added, which will increase the burden of the model. The convex optimization theory can effectively solve this problem. In addition to problem modeling, this paper has also solved an optimization problem to present performance results. The solution process has involved a number of convex optimization theories such as the transformation of non-convex problems, semidefinite relaxation and geometric programming. It can be seen that the convex optimization theory is of great significance to this paper (Bai and Zhu, 2021; Gupta and Sharma, 2021).

Convex set: If the line segment after connecting any two points in the set C is still in the set C, it satisfies:


(1) $$\forall {\text{x}}_{1},\forall {\text{x}}_{2}\in C,0\leq \theta \leq 1\to \text{x}=\theta \,{\text{x}}_{1}+\left(1-\theta \right)\,{\text{x}}_{2}\in \text{C}$$

Then the set C is convex.

Affine set: If the straight line passing through any two different points in the set C⊆R^n^ is still in the set C⊆R^n^, it works as follows:


(2) $$\forall {\text{x}}_{1},\forall {\text{x}}_{2}\in C,\theta \in \text{R}\to \text{x}=\theta \,{\text{x}}_{1}+\left(1-\theta \right)\,{\text{x}}_{2}\in \text{C}$$

Then the set C is an affine set.

Convex function: for a function


(3) $$f:{\text{R}}^{\text{n}}\to \text{R}$$

If the domain *dom*f is a convex set, and for ∀x,∀y ∈ *dom*f,0≤θ≤1, there is :


(4) f⁢(θ⁢x+(1-θ)⁢y)≤θ⁢f⁢(x)+(1-θ)⁢f⁢(y)

Then Equation 3 is called a convex function.

The first-order condition of a convex function: if the function is differentiable, the necessary and sufficient conditions for it to be convex are (Chan et al., 2022):


(5) ∀x,∀y∈dom⁢f

(6) $$f\,\left(y\right)\geq f\,\left(x\right)+\nabla f\,{\left(x\right)}^{\text{T}}\,\left(y-x\right)$$

∇⁡f(x)^T^ is the gradient of the function f within *dom*f.

Second-order conditions of convex functions: if the functionf is a second-order differentiable, then the necessary and sufficient condition for it to be a convex function is that its Hessian matrix is a positive semi-definite matrix. In other words, for ∀x ∈ *dom*f, there is,


(7) $$\nabla^{2}\text{f}\geq 0$$

When determining whether a function is a convex function, first-order and second-order conditions are usually used (Ma and Zhang, 2021; Zhao and Yang, 2021).

Affine function: If the function f has the form of a general equation,


(8) f⁢(x)=Ax+b

And the function does not go through the origin (in the case of b≠0), then the function is called an affine function. Among them, A, b can be expanded to: A ∈ R^m×n^, b ∈ R^m^.

Non-negative weighted summation: The non-negative weighted summation of a convex function is still a convex function. That means if f1,…,f_m_ is a convex function, and w_1_,…,w_m_ ≥ 0, then the following function is a convex function:


(9) $$\text{f}={\text{w}}_{1}\,{\text{f}}_{1}+\cdots +{\text{w}}_{\text{m}}\,{\text{f}}_{\text{m}}$$

Compound affine mapping: If the function f:R^n^→R, and A ∈ R^m×n^,b ∈ R^m^, the function g:R^m^→R is defined as:


(10) g⁢(x)=f⁢(Ax+b)

(11) domg={x|Ax+b∈domf}

At this time, if the function f is a convex function, the function g is a convex function. If the function f is a concave function, then the function g is a concave function.

Convex optimization problem: The standard optimization problem usually talked about has the following form:


(12) $$\,\text{minimize}\,{\text{f}}_{0}\,\left(x\right)\;\mathrm{subject}\,\mathrm{to}\,{\text{f}}_{\text{i}}\,\left(x\right)\leq 0,i=1,\cdots ,m\,{\text{a}}_{\text{i}}^{\text{T}}\,\text{x}={\text{b}}_{\text{i}},i=1,\cdots ,p$$

Among them, f_0_(x) is the objective function, f_i_(x)≤0,i=1,⋯,m is the inequality constraint, and ${\text{a}}_{\text{i}}^{\text{T}}\,\text{x}={\text{b}}_{\text{i}},i=1,\cdots ,p$ is the equality constraint. If f_i_(x) in the above formula are both convex functions and ${\text{a}}_{\text{i}}^{\text{T}}\,\text{x}={\text{b}}_{\text{i}}$ affine functions, the problem is called a standard form of convex optimization problem.

If f_0_(x), f_i_(x) and ${\text{a}}_{\text{i}}^{\text{T}}\,\text{x}$ in Equation 12 are all affine functions, the problem is called linear programming (LP) (Chen and Zhang, 2021; Liu et al., 2021). LP has the following form:


(13) $$\text{minimize}\,{\text{c}}^{\text{T}}\,\text{x}+\text{d}\;\mathrm{subject}\,\mathrm{to}\,\mathrm{Gx}\leq \text{h}\,\text{Ax}=\text{b}$$

G ∈ R^m×n^, and A ∈ R^p×n^. LP is a convex optimization problem. Because this article does not directly apply LP, and the LP solution is relatively simple, it will not be introduced in detail here.

Among the convex optimization problems of generalized inequalities, cone programming is the simplest, which consists of a linear objective function and an inequality constraint function. The function is affine as follows.


(14) $$\,\text{minimize}\,{\text{c}}^{\text{T}}\,\text{x}\;\mathrm{subject}\,\mathrm{to}\,\mathrm{Fx}+\text{g}\leq {\text{k}}_{0}\,\text{Ax}=\text{b}$$

When K is a non-negative quadrant, cone programming degenerates into linear programming. Cone programming can be understood as an extension of linear programming, by replacing the component inequalities in linear programming with generalized linear inequalities.

Semi-definite programming is also a generalization of linear programming. When K is a semi-positive definite matrix cone of k×k, the corresponding cone programming is called semi-definite programming. Semi-definite programming generally has the following form:


(15) $$\,\text{minimize}\,{\text{c}}^{\text{T}}\,\text{x}\;\mathrm{subject}\,\mathrm{to}\,{\text{x}}_{1}\,{\text{F}}_{1}+\cdots +{\text{x}}_{\text{n}}\,{\text{F}}_{\text{n}}\,\text{G}\leq 0\,\text{Ax}=\text{b}$$

Among them, G,F1,…,F_n_ ∈ S^k^ is the matrix cone of k×k, and A ∈ R^p×n^. The inequality constraint here is a linear matrix inequality. If G,F1,…,F_n_ are all diagonal matrices, the semidefinite program will degenerate into a linear program. Compared with the above forms, the standard form of semidefinite programming is more widely used:


(16) $$\,\mathrm{minimize}\,\mathrm{Tr}\,\left(\mathrm{CX}\right)\;\mathrm{subject}\,\mathrm{to}\,\mathrm{Tr}\,\left({\text{A}}_{\text{i}}\,X\right)\leq {\text{b}}_{\text{i}},i=1,\cdots ,p\,\text{X}\geq 0$$

Among them, C,A1,…,A_p_ ∈ S^n^, the matrix cone of n×n. For semidefinite programming, it can be solved directly through the CVX toolbox in the MATLAB platform.

If f_0_(x) in Equation (12) is a convex quadratic form, and f_i_(x) and ${\text{a}}_{\text{i}}^{\text{T}}\,\text{x}$ are both affine functions, the problem is called quadratic programming (QP). It is expressed as:


(17) $$\mathrm{minimize}\,\left(1/2\right)\,{\text{x}}^{\text{T}}\,\text{Px}+{\text{q}}^{\text{T}}\,\text{x}+\text{r}\,\;\mathrm{subject}\,\mathrm{to}\,\mathrm{Gx}\leq \text{h}\,\text{Ax}=\text{b}$$

$\text{P}\in {\text{S}}_{+}^{\text{n}}$, the positive semi-definite matrix cone of n×n, G ∈ R^m×n^*and*A ∈ R^p×n^. If the objective function is convex quadratic, the inequality constraints are also convex quadratic. For example,


(18) $$\,\mathrm{minimize}\,\left(1/2\right)\,{\text{x}}^{\text{T}}\,{\text{P}}_{0}\,\text{x}+{\text{q}}_{0}^{\text{T}}\,\text{x}+{\text{r}}_{0}\;\mathrm{subject}\,\mathrm{to}\,\left(1/2\right)\,{\text{x}}^{\text{T}}\,{\text{P}}_{\text{i}}\,\text{x}+{\text{q}}_{\text{i}}^{\text{T}}\,\text{x}+{\text{r}}_{\text{i}}\leq 0,i=1,\cdots ,m\,\text{Ax}=\text{b}$$

$\text{P}\in {\text{S}}_{+}^{\text{n}},\text{i}=1,\cdots ,m$. This problem is called QXQP. In the actual processing of QXQP, a new variable was added to replace the QXQP with a semidefinite program containing non-convex constraints, and then a semidefinite relaxation (SDR) is used to remove the non-convex constraints. This has converted the original problem into a convex semidefinite program. Then CVX toolbox on the MATLAB platform was invoked to solve.

## Impact of the blended teaching model of SPOC+PBL

**FIGURE 2:**
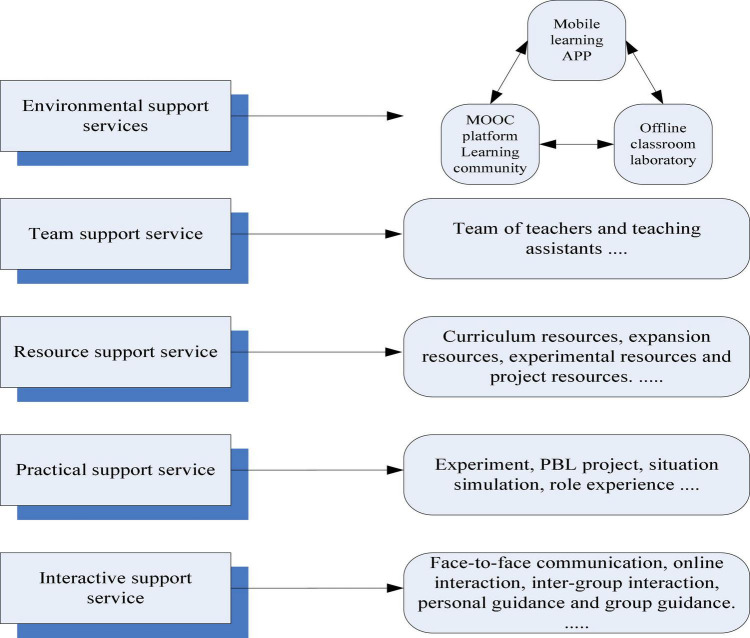
Learning support service design of flipped classroom teaching based on SPOC.

In order to understand the source of psychological pressure under the new model, this paper has set up a SPOC+PBL hybrid teaching model practice class for the course “English-Chinese Translation and Interpretation”. The overall situation of students is evaluated by a complete pre-class, in-class, and after-class system. Finally, this is the basis for the follow-up psychological stress model. The course design is shown in Figure 2.

### Preliminary preparation

The online learning platform of this course is Rain Classroom and Wisdom Tree Platform. Students can conduct online learning, online testing and progress inquiry on this platform, and teachers can use the background data to master students’ learning situation of English-Chinese translation and interpretation, thus realizing data-based teaching management.

The main teaching goal of this course is to cultivate students’ translation ability. On the basis of students’ clear understanding of the basic concepts of English-Chinese translation and interpretation, they can use their learned skills to think, analyze and solve practical problems in reality. Therefore, the teaching objectives of this course can be divided into three aspects-knowledge, ability and quality, as shown in Table 1.

**TABLE 1:** Course teaching objectives.

Primary target	Secondary target
Knowledge	Know and understand English-Chinese translation and interpretation
Ability	Familiar with the basic process of English-Chinese translation
Quality	Students can use their knowledge to solve practical translation problems

“English-Chinese Translation” rearranges the course content according to the actual teaching needs, and arranges 14 weeks of course teaching, including 7 online courses and 6 offline courses. In the offline courses, students are arranged to discuss in groups. The object of this survey is a total of 1,000 students in three grades, including 400, 400, 200 freshmen, sophomores and juniors, respectively. Through the students’ academic survey, it can be seen that the students participating in the course study are mainly concentrated in the second and third grades. Among them, 54.35% of the students have MOOC learning experience, and 47.83% of the students want to try new teaching forms. Although there are large individual differences among students in this course, students’ acceptance and expectation of the new teaching method can be seen. Teachers need to disassemble and repeatedly demonstrate the operation steps when teaching the experimental part of big data, and weaken the operation of some professional techniques on the basis of ensuring the integrity of the practice steps.

**FIGURE 3:**
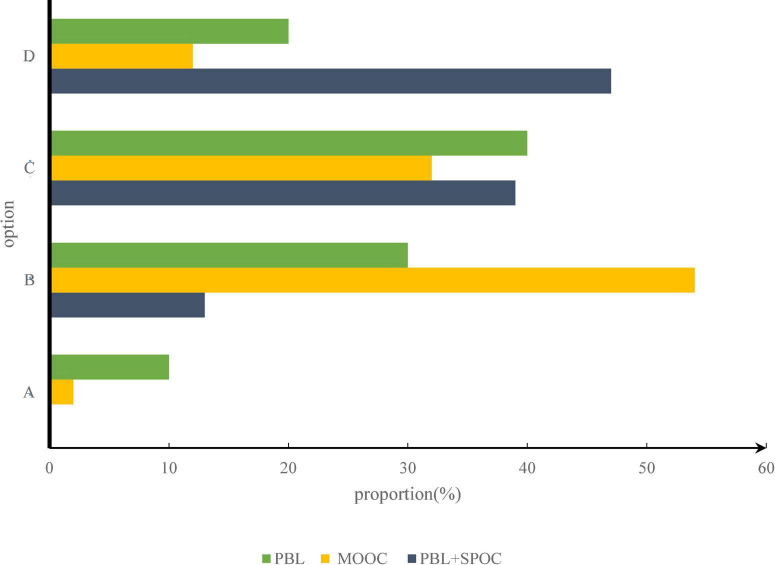
Students’ understanding of MOOC, PBL.

As shown in Figure 3, a means know very well, B means understand, C means heard, D means don’t understand. Through the investigation of students’ understanding of English-Chinese translation and interpretation, it can be found that 39.13% of students know something about English-Chinese translation and interpretation but don’t go deep into it, and only 13.04% of students are prepared to study English-Chinese translation and interpretation specifically, which indicates that students’ knowledge and skills about English-Chinese translation and interpretation are still in a state of learning, so teachers should pay attention to the professional level and presentation of theoretical knowledge in teaching.

### Teaching implementation process

#### Group learning before class

Students form groups freely. In the process of self-learning, students have various difficult questions. At this time, they can ask questions in the QQ learning community and ask for the help of teachers or classmates. Teachers need to answer the questions in time, discuss common problems raised by students, and record meaningful questions that can be learned by designing offline learning activities. At the same time, due to their own learning needs, some students can initiate temporary private conversations with teachers or teaching assistants in the QQ learning community to obtain targeted guidance and help.

#### Practice in class

There were 6 offline teachings in this course, including the pre-class tutorial class, the final report class and 4 face-to-face practical classes. Among them, the classroom content of the practical operation class was to disassemble the English-Chinese translation and interpretation experiment into special topics for study, and each experiment of the practical operation class was a part of the English-Chinese translation and interpretation project. In order to ensure that students with different professional backgrounds can learn technical operations, the experimental operation steps are simplified and easy to master. The teacher also strengthened the step-by-step disassembly demonstration of the experimental operation in the classroom.

#### Consolidation after class

After students were grouped, project-based learning was conducted in small groups. After discussion, each group first determined the topic of the project, which should have practical significance. An execution plan was then developed for the implementation of the project. The plan can be adjusted according to the actual situation of implementation, and finally through the division of roles within the group, group project collaboration was carried out. In the end, the group worked together to complete a English-Chinese translation and interpretation project based on real problems. In the whole process of project-based learning, teachers need to provide corresponding learning support to students’ learning needs. In the problem determination part, the teacher has guided the feasibility and technicality of the topic. As for project planning, teachers helped students sort out and improve the execution of projects. As for the project implementation section, teachers provided support for the theoretical techniques required in the project implementation process.

#### Multiple evaluation

The final assessment of college courses generally includes normal assessment and final examination, but the final assessment has been adjusted in this course. In order to highlight the emphasis on the learning process in the flipped classroom teaching based on SPOC, the proportion of the usual academic performance has been increased in the final assessment, and the final examination has been weakened. At the same time, the usual assessment was divided into online learning assessment, learning task assessment and team assessment project evaluation, as shown in Table 2. In the experiment of this paper, students’ performance will be evaluated through the scoring rules in Table 2, and this evaluation will be used as the effect of students’ learning.

**TABLE 2:** Criteria for student grading.

Project	Category
E-learning	Video progress is over 90%
	Video progress between 89% and 80%
	Video progress is below 80%
Learning assignment	6 must-do tasks
	6 optional tasks
Team project	Teacher rating 60%
	Student Grade 30%
	Personal project report 10%
Final exam	Combination of paper examination and computer examination

### Results of blended classroom practice

**FIGURE 4:**
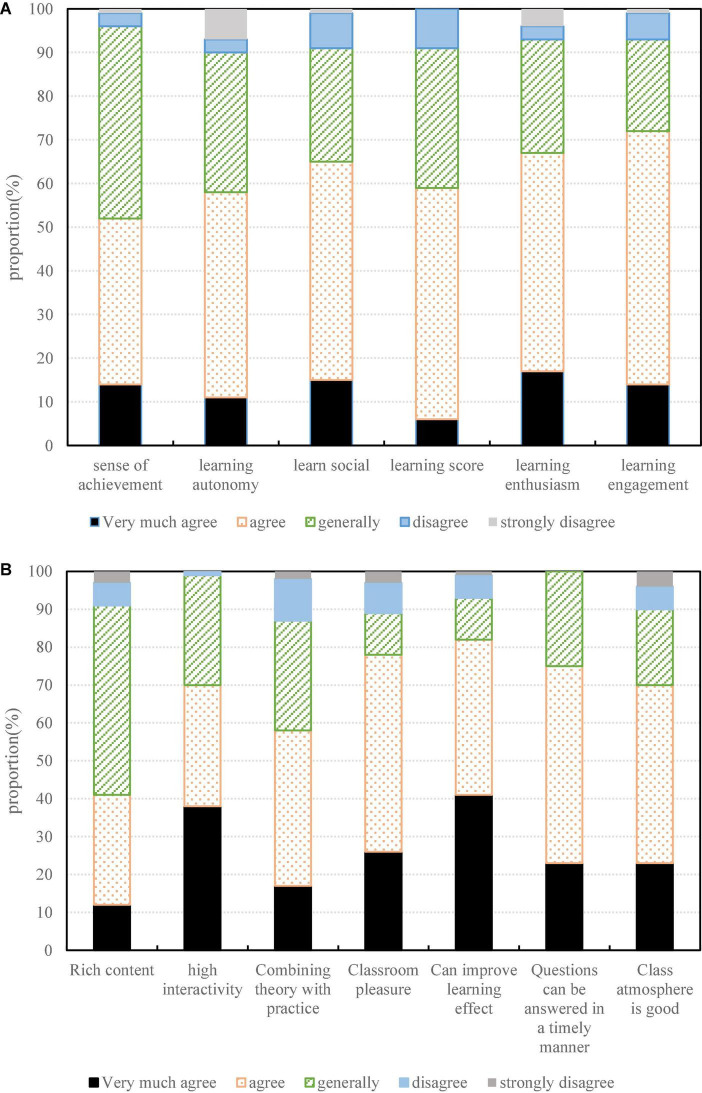
Effect of classroom practice. **(A)** Learning effect feedback. **(B)** Teaching mode feedback.

The learning effect is mainly based on the students’ personal subjective feelings. According to the factors that affect the individual’s learning effect, sense of achievement, autonomy, participation, sociality and teamwork, six survey questions have been designed, and five options have been set based on the five-level principle of Likert scale. The results of the investigation on the learning effect are shown in Figure 4A. It can be seen from the figure that in terms of learning achievement, learning autonomy, learning participation, and learning interaction, more than half of the students indicated that they had a good subjective learning effect, and believed that their internal motivation was satisfied in the learning process. In terms of team division of labor, 67.65% of the students indicated that they had improved their ability to analyze and solve problems during this learning process. In terms of teamwork, 73.53% of the students indicated that they had improved their teamwork problem-solving ability during this learning process. According to self-determination theory, the intrinsic motivation of individual students can bring better persistence, performance and satisfaction to the individual than extrinsic motivation. It can be found that in this learning process, most of the students have a good learning effect, which has also showed that the students have a good subjective learning experience in the learning. It has also showed from the side that giving students independent learning space, enhancing the communication between teachers and students, students and students, and carrying out project-based learning are beneficial to improve students’ learning effect from the internal motivation. However, learning activities should be regulated according to the actual situation, and attention should be paid to maintaining students’ continuous participation in learning on the basis of stimulating students’ learning from internal motivation.

As can be seen from Figure 4B, 76.47% of the students surveyed expressed that they were satisfied with the overall feeling of the study and the learning effect obtained, and 70.59% of the students indicated that they would recommend or continue to participate in this form of learning where there is an opportunity. Therefore, it can be seen that students have high learning satisfaction and model recognition for the SPOC-based flipped classroom. Although there are still more or less problems in the current learning process, which need to be improved in future teaching, in general, this teaching mode has been recognized and accepted by students, and has value of continuous use and promotion.

## Psychological stress factors of mixed teaching mode

### Establishment of pressure model

According to expectation theory, individuals usually weigh risks and benefits before making decisions. When the possibility of making a decision is more likely to bring benefits than risks, individuals will actively make decisions; on the contrary, individuals will reduce the possibility of decision-making, even stop making decisions. Then students will evaluate the risks and benefits they generate when making decisions about psychological counseling. If we can reduce students’ risk perception, then we can improve students’ decision-making behavior intention of psychological counseling. So as to improve the possibility of professional psychological consultation. For the school leadership, it is unrealistic to directly force students and teachers to conduct professional psychological counseling, and the leadership cannot directly define whether their state is within the controllable range through the performance of students and teachers, so the psychology of students and teachers needs to be evaluated by themselves. When students and teachers assess that they need psychological counseling, their behavior intention of psychological counseling can be influenced. Therefore, starting from their behavior intention, improving their behavior intention of psychological counseling can effectively increase the possibility of psychological counseling between students and teachers, thus promoting professional psychological counseling between students and teachers, thus achieving the goal of psychological counseling.

This paper has divided the factors that affect psychological stress behavior intention into six factors, including “self-esteem strength”, “self-efficacy”, “help-seeking experience”, “psychological counseling cognitive bias”, “counseling object positioning bias” and “the availability of psychological counseling resources “. These six factors can directly affect the behavioral intention of students’ psychological counseling, but whether they are related to risk perception needs to be verified.

### Experimental data analysis

#### Sample and data collection

The data of this study were obtained through a questionnaire survey. A total of 250 questionnaires were distributed and 234 were recovered. After excluding invalid questionnaires, 212 valid questionnaires remained, with a recovery rate of 84.80%. Independent samples *T* test was used to analyze whether there were significant differences in the grades and genders of the questionnaire samples. The results showed that *t* > 0.05 was not significant, indicating that there was no response bias in the samples. In this paper, the questionnaire is randomly arranged to reduce the common method bias, and irrelevant questions are inserted between the variable questions to measure whether the subjects fill in carefully. All variables were measured on a 7-point Liker scale, with 1 indicating “completely disagree” and 7 indicating “completely agree”.

**FIGURE 5:**
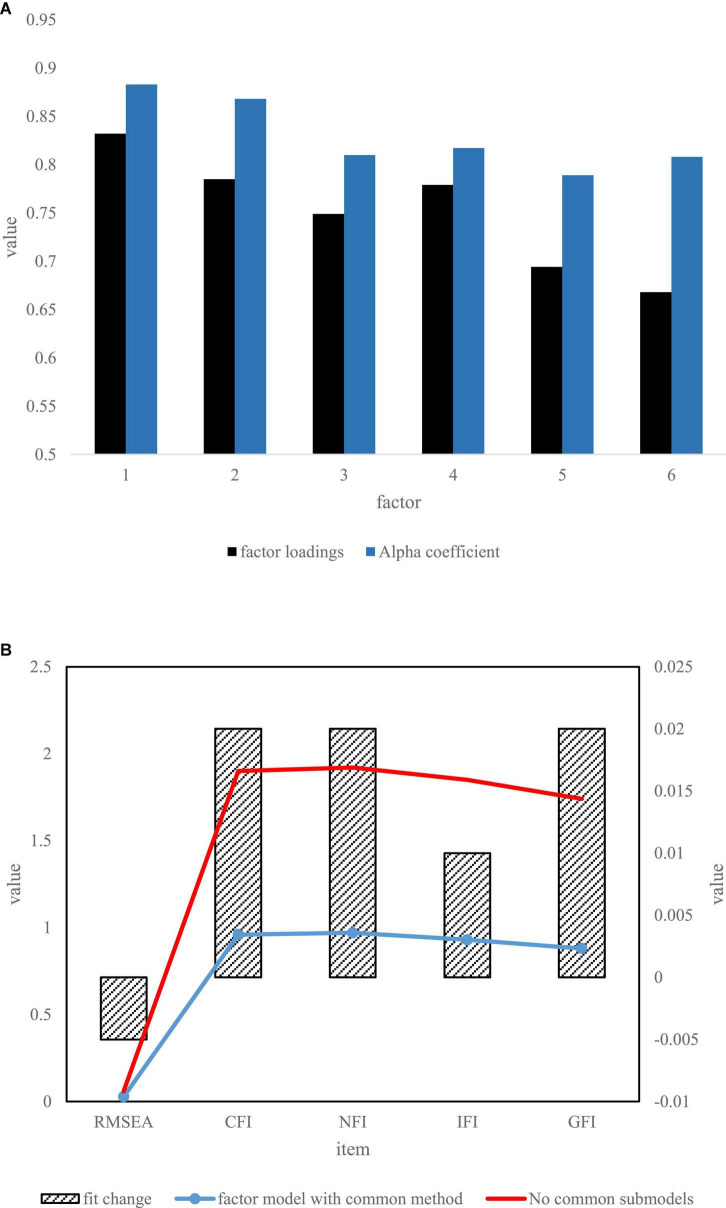
Reliability of questionnaire result. **(A)** Results of Reliability and validity destruction. **(B)** Results of common method bias test.

In the experiment, SPSS software was used to analyze the data, as shown in Figure 5. For the reliability of the six factors, the Cronbach α coefficients were all greater than 0.5, the reliability of the variable was up to about 0.88, and the highest efficiency of the variable was about 0.83. It has been shown that the factors selected in this experiment are compound standards, and the results of the common method deviation test have also shown that the fitting of the samples is good, and the theoretical model can be supported in general.

### Model verification and results

**FIGURE 6:**
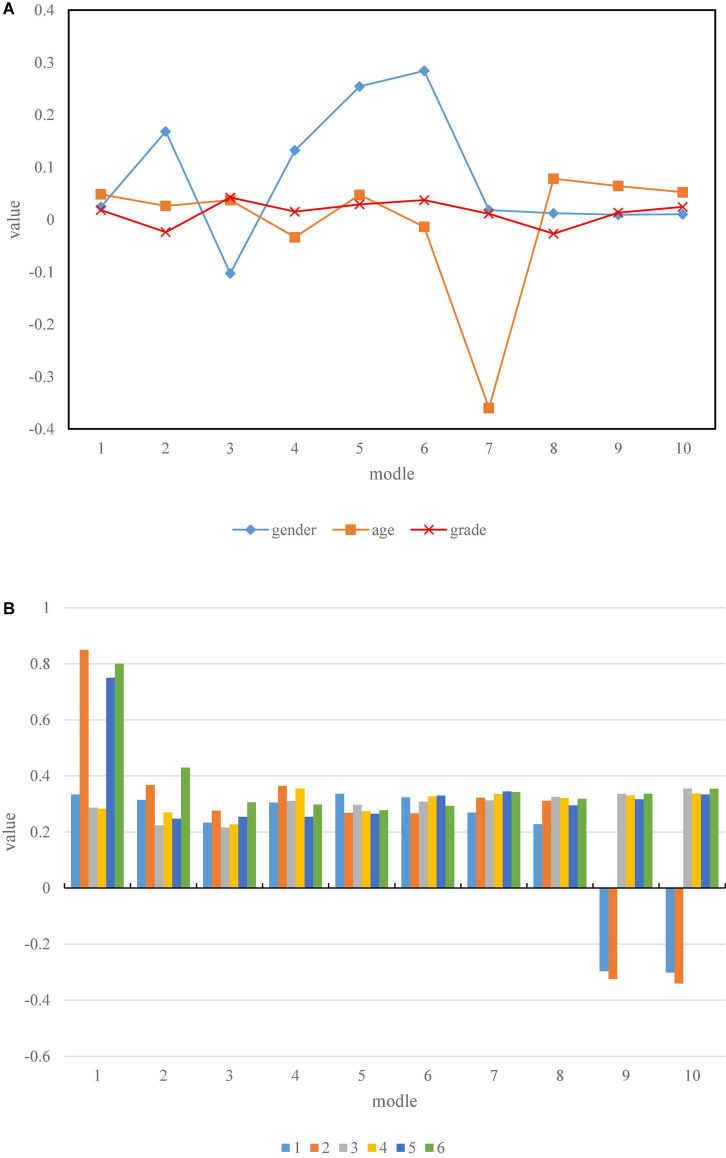
Model destruction results. **(A)** Results of basic regression. **(B)** Regression of 6 factors.

The influencing factor is the independent variable, the risk perception is the mediating variable, the concealment of personal information in telepsychological counseling is the moderating variable, and the students’ psychological behavioral intention is the basic model. The specific steps to test the second half of the mediation path of the hidden moderation of personal information in remote psychological counseling include: (1) the regression of students’ psychological counseling behavior intention to the impact factor and the concealment of personal information of remote psychological counseling; (2) the regression of risk perception to the influence factors and the concealment of personal information in remote psychological counseling; (3) the regression of students’ psychological counseling behavior intention to influencing factors, the concealment of personal information of remote psychological counseling, and risk perception; (4)the regression of behavioral intention of students’ psychological counseling to influencing factors, the concealment of personal information in remote psychological counseling, risk perception, risk perception × the concealment of personal information in remote psychological counseling. If the coefficients of the influencing factors in the first two steps are significant, the risk perception coefficient in the third step is significant, and the concealment coefficient of risk perception × remote psychological counseling personal information in the fourth step is significant, then the moderated mediating effect is established. At the same time, in order to reduce the multicollinearity problem between variables in the regression equation, it is necessary to centralize and standardize the independent variables and moderator variables before constructing the product term, and then inherit the processed independent variables and moderator variables. In this study, the standardized processing method in SPSS software has been selected. The research results are shown in Figure 6.

As shown in Figure 6, the comparison between the groups before the experiment: the psychological stress levels of the two groups conform to a normal distribution, and the age, gender, and grade are not significantly different. The independent samples *t* test showed that *P* = 0.365, 0.098/0.125 > 0.05, the difference was not statistically significant, and it was comparable.

After the experiment, the comparison between the two groups: the psychological stress level of the control group after the experiment conformed to the normal distribution, and the paired *t* test showed that *P* = 0.013, 0.024 < 0.05, and the difference was statistically significant, indicating that the control group was effective in relieving the stress level; the experiment After treatment, the 6-factor levels in the group were in line with normal distribution, and *P* = 0.000, 0.004 < 0.05 were obtained by paired *t* test for comparison within the group, and there were significant differences, indicating that the student psychological counseling intervention program was effective in relieving psychological pressure.

## Conclusion

After theoretical deduction and empirical analysis, the main conclusions are as follows. (1) Students’ self-esteem, help-seeking experience and psychological counseling cognitive biases have significant effects on the risks of stigma, expected utility of self-disclosure and expected self-disclosure. (2) Students’ self-efficacy, counseling object orientation bias and the availability of psychological counseling only had a significant effect on the risk of utility, expected self-disclosure and its perception. (3) The risk of expected utility of self-disclosure has the greatest influence on students’ psychological counseling behavior intention. (4) The concealment of personal information in remote psychological counseling can reduce the risk of stigma, expected utility of self-disclosure and the perception of expected risk of self-disclosure, and then increase the behavioral tendency of students in psychological counseling.

## Data availability statement

The original contributions presented in this study are included in the article/supplementary material, further inquiries can be directed to the corresponding author.

## Author contributions

GS designed the work and drafted and finalized the manuscript. YA collected and analyzed the data. Both authors approved the final version of the manuscript.
